# Exploitation of Unconventional CD8 T-Cell Responses Induced by Engineered Cytomegaloviruses for the Development of an HIV-1 Vaccine

**DOI:** 10.3390/vaccines13010072

**Published:** 2025-01-14

**Authors:** Joseph Bruton, Tomáš Hanke

**Affiliations:** 1 Hertford College, University of Oxford, Oxford OX1 3BW, UK; joseph.bruton@hertford.ox.ac.uk; 2 The Jenner Institute, Nuffield Department of Medicine, University of Oxford, Oxford OX3 7DQ, UK

**Keywords:** cytomegalovirus, HCMV, RhCMV68-1, HLA-E, CD8 T-cells, HIV-1 vaccines, vaccines, SIV challenge, protection, clearance, intracellular trafficking, MHC-E, rhesus macaques

## Abstract

After four decades of intensive research, traditional vaccination strategies for HIV-1 remain ineffective due to HIV-1′s extraordinary genetic diversity and complex immune evasion mechanisms. Cytomegaloviruses (CMV) have emerged as a novel type of vaccine vector with unique advantages due to CMV persistence and immunogenicity. Rhesus macaques vaccinated with molecular clone 68-1 of RhCMV (RhCMV68-1) engineered to express simian immunodeficiency virus (SIV) immunogens elicited an unconventional major histocompatibility complex class Ib allele E (MHC-E)-restricted CD8^+^ T-cell response, which consistently protected over half of the animals against a highly pathogenic SIV challenge. The RhCMV68-1.SIV-induced responses mediated a post-infection replication arrest of the challenge virus and eventually cleared it from the body. These observations in rhesus macaques opened a possibility that MHC-E-restricted CD8^+^ T-cells could achieve similar control of HIV-1 in humans. The potentially game-changing advantage of the human CMV (HCMV)-based vaccines is that they would induce protective CD8^+^ T-cells persisting at the sites of entry that would be insensitive to HIV-1 evasion. In the RhCMV68-1-protected rhesus macaques, MHC-E molecules and their peptide cargo utilise complex regulatory mechanisms and unique transport patterns, and researchers study these to guide human vaccine development. However, CMVs are highly species-adapted viruses and it is yet to be shown whether the success of RhCMV68-1 can be translated into an HCMV ortholog for humans. Despite some safety concerns regarding using HCMV as a vaccine vector in humans, there is a vision of immune programming of HCMV to induce pathogen-tailored CD8^+^ T-cells effective against HIV-1 and other life-threatening diseases.

## 1. Introduction

Although combination antiretroviral therapy (cART) has transformed AIDS from a fatal disease into one associated with a near-normal lifespan and quality of life, regrettably cART does not eliminate the virus from the body and, therefore, is life long. Over 39 million people live with HIV-1, the highest number in the history of the pandemic, of whom an estimated 76% received cART in 2022 [1] missing the UNAIDS-targeted goals. The development of a prophylactic HIV-1 vaccine remains an unmet global health need; however, finding one has been challenging mainly due to HIV-1’s enormous genetic diversity [2,3] and complex mechanisms of immune evasion [4,5]. Over the past four decades, a handful of strategies have been tested unsuccessfully in clinical trials for their preventive efficacy against HIV-1. These strategies employed recombinant proteins and genetic subunit vaccines vectored by plasmid DNA as well as engineered replication-deficient avian poxvirus ALVAC or human adenoviruses and their heterologous prime–boost combinations, and aimed to induce protective antibodies and T-cells [6,7,8,9,10]. Where traditional vaccine approaches are unsafe or failing, alternative vaccine strategies that elicit unconventional immune responses which pathogens have not evolved to avoid may come to the rescue.

In this respect, the use of cytomegaloviruses (CMV) as a vaccine vector has recently garnered significant attention due to the unique CMV’s immunogenicity, its life-long persistence and its ability to elicit distinct patterns of T-cell responses when genetically manipulated. Remarkably, the deletion of specific genes in rhesus macaque CMV during the generation of molecular clone 68-1 (RhCMV68-1) and the expression of simian immunodeficiency virus (SIV) immunogens was able to control pathogenic SIV replication in over half of the challenged rhesus macaques [11,12]. Although RhCMV68-1 did not prevent the acquisition of the challenge SIV, it arrested SIV replication and cleared the virus from the protected animals, which appeared functionally cured [11,12,13,14,15]. The expressed transgene products included either SIV Gag, Rev-Tat-Nef, Protease-Reverse Transcriptase of Pol or six-conserved Gag/Pol regions called SIVconsv239 as concatenated polyproteins ([11,12,13,14,15] and TH, Picker et al. unpublished). Notably, no surface glycoprotein Env was present in the vaccines, and no SIV-specific antibodies were induced or involved in the protection. This was accompanied by RhCMV68-1-induced unconventional transgene product-specific CD8^+^ T-cell responses restricted by non-classical major histocompatibility complex (MHC) class Ib allele E (MHC-E) and MHC class II (MHC-II) but not by canonical MHC class Ia alleles A, B, C [11,12,16]. This observation pointed to a previously unidentified Achilles’ heel of HIV-1, which engineered CMV vectors could exploit [17]. The publication of these results generated great excitement, expectations and scientific curiosity.

This article highlights several features of the biology underlying CMV vectors along with the current understanding of MHC-E immunological functions and intracellular trafficking. It discusses the challenges of translating the protection observed in macaques to humans for developing prophylactic and therapeutic vaccines against HIV-1 as well as the initial attempts to construct human CMV (HCMV)-based vaccine candidates. A successful translation to humans would represent a significant advancement in HIV-1 treatment and prevention.

## 2. CMVs as a Vaccine Vector

CMVs are ubiquitous β-herpesviruses with human seroprevalence ranging from 50% to 90%, primarily depending on geographic region, age and socioeconomic conditions [18]. HCMV causes life-long asymptomatic infection in healthy individuals and has evolved mechanisms to evade certain aspects of the immune system while strongly activating others, generating sustained immune responses without overwhelming disease in these individuals. Due to these unique immunological properties, HCMV is being exploited as a vaccine vector [17,19,20,21]. The main advantages include its persistence and the induction of robust, life-long T-cell responses with an expanded functional unexhausted T-cell memory maintained by periodic reactivations [22,23]. Because of their placement in tissues throughout the body, such T-cells could mediate immediate immune interception of nascent HIV-1 infection [24]. These features could be harnessed for the next generation of vaccines to tackle challenging infectious diseases such as HIV-1/AIDS and certain cancers. A few examples of using CMVs include an engineered murine CMV inducing *Mycobacterium tuberculosis* (Mtb) antigen 85A-specific T-cells that provided protection against Mtb in a mouse model [25]. A reduction in infection by 68% compared to unvaccinated animals was reported in rhesus macaques using RhCMV68-1 expressing Mtb antigens [26]. Additionally, RhCMV68-1-vectored vaccines significantly reduced the release of liver stage parasites of *Plasmodium knowlesi* into the blood [27] and cynomolgus CMV (CyCMV)-based vaccines protected macaques against an influenza virus challenge [28]. Regression of solid tumours was linked to a CMV-derived vector that elicited CD8^+^ T-cell memory inflation [29,30], that is a gradual accumulation of large populations of functional effector and effector-memory cells. Examples of vectored tumour-associated antigens include retinoic acid early induced transcript-1γ (RAE-1γ), tyrosinase-related protein 2 (TRP2) and melanoma antigen gp100/PMEL [31,32,33].

## 3. RhCMV68-1 Arrests SIV Replication

In a typical experiment [11,12,13,14,15], Hansen, Picker, Früh and colleagues vaccinated rhesus macaques twice 14–18 weeks apart with an RhCMV68-1 vaccine expressing SIV immunogens. One year later, the animals were repeatedly challenged with highly pathogenic SIVmac239 via the intrarectal or intravaginal route until the SIV uptake was documented through detecting the SIV genomes by PCR. Approximately 59% of the vaccinated and challenged animals exhibited an immune-mediated SIV replication arrest, while the infected animals were very similar to unvaccinated controls [13]. All protected animals became infected and the presence of SIV in the tissues was initially detected; however, further virus spread from the port of entry was prevented, the titres declined over the first 8–12 weeks and, eventually, by week 20, SIV was cleared from the body [12,14]. The protected macaques were no longer distinguishable by immunological or virological criteria from never-challenged [uninfected] animals [11,12,13,14,15]. These macaques appeared functionally cured of SIV, indicating that the virus was undetectable in the absence of antiretroviral treatment. When the long-term-protected animals were necropsied, extensive tissue analysis using ultrasensitive assays detected no SIV RNA/DNA in most specimens, and no infectious virus was demonstratable by adoptive transfer of peripheral blood mononuclear cells (PBMC) to naïve macaques, which represents the most sensitive test for the presence of an infectious virus within the cells [12,14]. In the protected animals, the vaccine-elicited CD8^+^ MHC-E-restricted T-cells were maintained for long periods, and 80% of these protected/cured monkeys could clear SIV when rechallenged up to 10 years later [12,14]. The same vaccine did not work therapeutically when administered to animals that received antiretroviral treatment 4–9 days after infection [34]. The mechanism of protection was consistent with immune-mediated virus replication arrest distinct from the previously described elite HIV-1/SIV control [35,36,37,38,39]. Finally, it was shown that RhCMV68-1-vectored vaccines are not hampered by pre-existing immunity in already RhCMV-infected animals [11]. This would be an important vaccine feature if this alternative CD8^+^ T-cell-based HIV-1 vaccine was to be used in humans.

## 4. Unconventional CD8^+^ T Cells Induced by RhCMV68-1

Since RhCMV vectors elicit little to no antibodies against the transgene products, the observed protection is likely due to cellular immunity. However, RhCMV68-1-induced CD8^+^ T-cells were unconventional and failed to recognise canonical epitopes presented by MHC-Ia, and instead recognised multiple MHC-II and MHC-E epitopes [11,12,13,14,15]. Notably, all immunised animals recognised so-called universal MHC-E and MHC-II supertopes [11,12,13,14,15]. CD8^+^ T-cell depletion using anti-CD8β antibodies abrogated the protection confirming that these cells are a major component of the defence [17]. The MHC-E and MHC-II CD8^+^ T-cell responses were similar in magnitude, functionality, memory subtypes and breadth of epitope recognition [11,12,13,14,15]. Elegant investigations using inhibitory tissue-specific micro RNAs (miRNA) to abrogate productive RhCMV68-1.SIV infection in specific cell types allowed for the separation of the induction of MHC-E from MHC-II CD8^+^ T-cells [13,15,40], and it was the MHC-E-restricted CD8^+^ T-cells that were necessary and sufficient for RhCMV68-1-mediated protection against SIV [13,41]. This could have been achieved not only with full-length SIV proteins and conserved sub-protein regions, but also with a supertope-focused transgene, suggesting a focused SIV sequence targeting [16,42].

## 5. Protection Was Associated with Upregulated IL-15 Transcription Program

There remained still at least one major puzzle to solve: both the protected and unprotected animals exhibited MHC-E CD8^+^ T-cells, which were functionally and phenotypically inseparable. This pointed to the significant involvement of other factors [13]. When longitudinal whole-blood transcriptomic analysis was performed to search for associations between immune signatures and challenge protection, it highlighted the increased post-vaccination transcriptional activity of genes regulated by interleukine (IL)-15 signalling, which were inversely correlated to their pre-vaccination levels [41]. IL-15 regulates effector memory T-cell physiology and may influense MHC-E-restricted CD8^+^ T-cells by promoting differential migration, enhancing effector functions, and modulating epitope sensitivity or TCR-triggering signal threshold [41]. Thus, the unconventional CD8^+^ T-cell response can be either directly fine-tuned by the IL-15 immune signature or functionally facilitated by its effect on other cell types. A separate study involving 45 RhCMV68-1.SIV-vaccinated rhesus macaques discovered a significant correlation between the gut microbiome, early vaccine-induced immune signatures and the challenge outcome [43]. Consequently, a better understanding the IL-15’s role and the gut microbiome’s impact on protection will guide the design of effective CMV-based vaccines.

## 6. MHC-E as a Link Between Innate and Adaptive Responses

MHC-E is a highly conserved, non-classical MHC-Ib molecule that plays crucial roles in immune regulation. It helps maintain immune balance by protecting healthy cells from attacks by natural killer (NK) cells and is involved in CD8^+^ T-cell immune responses against infections and cancer. MHC-E is ubiquitously expressed on somatic cells, although, without activation, at lower levels than classical MHC-Ia molecules [44]. It is virtually non-polymorphic, with the human leukocyte antigen (HLA) existing in just two forms, HLA-E*01:01 and HLA-E*01:03. These variants differ by a single amino acid at position 107 (arginine and guanine, respectively) located outside the peptide binding groove on a loop between β-strands in the α_2_ domain of the heavy chain [45]. Of the two, HLA-E*01:03 tends to be more stable, resulting in a moderately higher surface expression, which may lead to a different impact on immune response and NK regulation compared to HLA-E*01:01.

The molecular basis for NK cell self-tolerance involves inhibitory signals that recognise self-MHC-Ia molecules primarily through natural-killer-cell and leukocyte immunoglobulin-like receptors KIR and LILR, respectively. Additionally, the interaction of MHC-E molecules loaded with VMAPRTL(L,I,V,F)L peptides (VL9), which are derived from the leader sequences of the MHC-Ia heavy chains, occurs with inhibitory CD98/NKG2 receptors [46,47]. While virus-infected cells are most effectively killed by CD8^+^ cytotoxic T-cells that recognise viral [foreign] peptide-loaded MHC-Ia, many viruses (and cancers) have evolved to downregulate surface MHC-Ia to shield the virus factories from CD8^+^ T-cells [48]. Indeed, the HCMV unique short genome segment US2 and US11 gene products downregulate classical HLA-Ia molecules to evade cytotoxic CD8^+^ T-cell responses [49]. MHC-E remains resistant to downregulation and, in addition to presenting MHC-Ia-derived VL9 peptides, can also present microbial peptides to CD8^+^ T-cells [50,51,52]. In this respect, CMV encodes its own identical VL9 peptide, derived from the Rh67/UL40 gene product, to sustain HLA-E trafficking and enhance surface expression of HLA-E thereby avoiding NK cell killing [42,53].

## 7. Benefits of the MHC-E-Restricted CD8^+^ T-Cell Protection

Vaccine-induced HLA-E-restricted CD8^+^ T-cell responses against HIV-1 offer several potential advantages. Although certain pathogens such as Mtb induce robust HLA-E-restricted CD8^+^ T-cells that are specific for at least 36 Mtb peptides [50,51,52], natural infections with HIV-1 and SIV do not prime MHC-E-restricted CD8^+^ T-cells to any appreciable, that is biologically significant levels. Consequently, HIV-1 has likely never been under selective pressure to develop strategies for avoiding HLA-E presentation. Furthermore, HIV-1 does interfere with HLA-Ia presentation, and the retained, potentially even slightly enhanced levels of viral peptide-loaded HLA-E molecules on the surface of infected cells provide an appealing vaccine target for the CD8^+^ T-cell killers [54]. Given the lack of HLA-E polymorphism, effective HLA-E-restricted CD8^+^ T-cells would benefit all individuals regardless of their HLA-Ia haplotypes thereby simplifying the vaccine design—if such CD8^+^ T-cells can be induced by vaccination [54].

## 8. Unusual Intracellular Trafficking, Surface Expression and Structure of MHC-E

**Figure 1:**
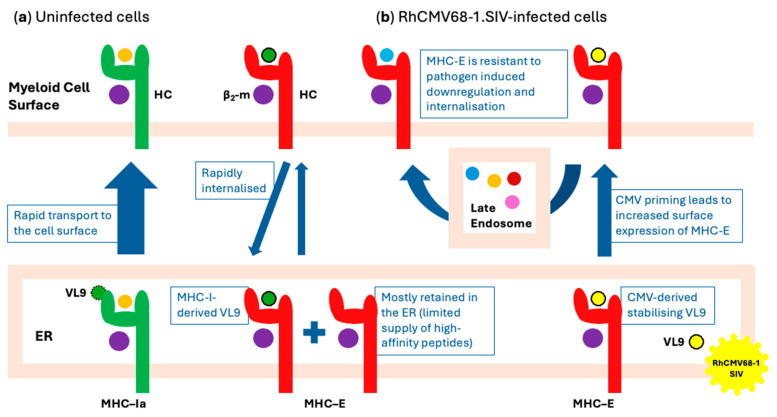
Suggested intracellular trafficking of HLA-E in myeloid cells. (**a**) Relative transport efficiency of classical MHC-Ia and MHC-E between the ER and cell surface. (**b**) One possible pathway for loading MHC-E with microbial peptides in RhCMV68-1-infected myeloid cells. HC—heavy chain of HLA-Ia (green) and HLA-E (red); β_2_-m—β_2_-microglobulin; ER—the endoplasmic reticulum; VL9—peptide derived from HLA-Ia (green) and CMV (yellow).

Developing a successful HIV-1 vaccine that utilises MHC-E-restricted CD8^+^ T-cells necessitates an understanding of MHC-E’s intracellular transport to the cell surface and recycling, the site(s) of the peptide cargo loading or exchange, and surface stability. After synthesis and peptide loading, typical classical MHC-Ia molecules rapidly exit the endoplasmic reticulum (ER) and reach the cell surface. In contrast, MHC-E is largely retained in the ER due to a limited supply of high-affinity peptides [55]. Once on the cell surface, MHC-E is unstable and is quickly internalised into late, recycling endosomes guided by its cytoplasmic tail [55] (Figure 1a). Natural HIV-1 and SIV infections do not prime MHC-E-restricted T-cells because of the limited presence of MHC-E on the CD4^+^ T-cell surface and possibly a lack of other costimulatory molecules. Indeed, the infection of myeloid cells (macrophages and dendritic cells) by the RhCMV68-1 vaccine was necessary for the induction of MHC-E-restricted CD8^+^ T-cells and for the protection of half of the challenged rhesus monkeys [13]. In infected macrophages, RhCMV68-1 priming ensures transporter associated with antigen processing (TAP)-independent MHC-E loading and simultaneously provides its own VL9 to stabilise MHC-E. It has been demonstrated that the expression of RhCMV VL9 peptide was necessary for priming the MHC-E-restricted SIV-specific CD8^+^ T-cell responses by RhCMV68-1.SIV [53].

The unusual intracellular traffic of MHC-E partly explains the induction of unconventional immune responses. Firstly, MHC-E is relatively resistant to additional surface downregulation. Pathogens downregulate the surface expression of classical MHC-Ia molecules by blocking peptide loading, and inhibiting the proteasome, TAP and other chaperones [56]. Because MHC-E is already retained in the ER, this does not further reduce its surface levels [55]. Similarly, pathogens, including HIV-1, often internalise MHC-Ia molecules by targeting the MHC-Ia cytoplasmic tail [55,57], but this does not affect MHC-E, which already undergoes rapid internalisation through this mechanism [55]. Additionally, some pathogens such as Mtb and *Salmonella typhi* provide MHC-E-binding peptides to enhance MHC-E surface expression and evade NK cell killing [51,58,59].

The structure of MHC-E is unusual. The human HLA-E molecules differ from classical HLA-Ia in five amino acid positions within the binding groove, which together optimise the binding of the VL9 peptides [60,61]. Note, however, that while the VL9 peptides are the dominant MHC-E binders, their affinities for the MHC-E groove are still lower than those of average peptides binding to the classical MHC-Ia molecules and does not significantly stabilise the MHC-E on the cell surface [62,63,64]. Nevertheless, the binding of ‘low-affinity’ VL9 results in a compact HLA-E structure [64,65] sufficient to pass the quality checks for ER exit [66]. Endogenous MHC-Ia-derived VL9 peptides do not saturate MHC-E in the ER [55] and it remains to demonstrate whether the HCMV VL9 does. Another distinction from MHC-Ia molecules is that at least in vitro, empty MHC-E heavy chains without any peptide remain associated with β_2_-microglobulin and stay open and peptide-receptive [65]. This may be more relevant for late endosomes, where the tail-recycled HLA-E can be reloaded with late endosome peptides and recycled to the surface [55,67] (Figure 1b). Whether the VL9 peptides dissociate from MHC-E on the cell surface or in the endosomes is a subject of investigation. Finally, it is encouraging that natural wild-type HCMV infection induces HLA-E-restricted CD8^+^ T-cells, which can recognise the HLA-E-presented UL40-derived VL9 peptide and kill HCMV-infected cells [68,69] demonstrating that there are sufficient VL9-loaded HLA-E molecules on the cell surface for the CD8^+^ T-cells to exert their effector function.

## 9. The Genetic Makeup of RhCMV68-1 and Translation to Humans by Orthology

The unconventional CD8^+^ T-cell response was attributed to specific RhCMV68-1 gene deletions of macaque orthologs of the human UL128/UL130 and UL146/UL147 genes that occurred during in vitro passaging to establish the RhCMV68-1 molecular clone [70]. This affected the pentameric complex of CMV on the virus envelope that is crucial for the virus’s ability to infect epithelial and endothelial cells. In this complex, three unique large (UL) genome segment open-reading frames UL128/UL130/UL131a encode pentameric complex subunits that bind to tissue-specific cell-surface receptors. Glycoproteins H and L (gH and gL) form the core of the complex and are essential for the virus to fuse with the host cellular membrane and enter the cell. Also other known viral proteins, likely along with some yet unidentified ones, are involved (or need to be absent) for the fine-tuning of intracellular trafficking and peptide loading of HLA molecules leading to the induction of HLA-E-restricted CD8^+^ T-cells and the SIV arrest [53,71].

CMVs are highly species-adapted viruses. The vaccine-design-by-orthology approach, that is manipulating functional gene analogues, has yielded some results in closely related Mauritian-cynomolgus macaques [15,72]. The deletion of the CyCMV orthologs of the RhCMV68-1 missing genes induced mixed MHC-E and MHC-II-restricted CD8^+^ T-cell responses with no MHC-Ia responses, reproducing the same replication arrest protection predicted by the IL-15 gene expression pattern [15,72]. Similarly to RhCMV68-1.SIV-vaccinated cynomolgus macaques, engineered CyCMV.SIV failed to protect rhesus macaques, reinforcing the concept of species adaptation. The challenge remains to translate these biological mechanisms to a much more evolutionary distant HCMV for human use.

To reproduce the macaque results in humans, at least two conditions must be met: HCMV must be engineered to successfully mimic the RhCMV68-1 vector, and the human immune response must utilise the same processes as are involved in macaques. There is a significant difference between the macaque and human immune systems, particularly concerning the MHC complexes, which are far more polygenic and polymorphic in monkeys [73,74]. This could alter thymic selection, making the immune repertoire of monkeys more prone to the observed unorthodox responses. Moreover, due to long-term coevolution with humans, HCMV is significantly more divergent than other nonhuman CMV species, especially in genes encoding immunomodulatory proteins [75]. These differences were highlighted by the experimental Towne/Toledo chimeric HCMV virus, which, despite lacking the pentameric complex, elicited completely canonical HLA-Ia-restricted CD8^+^ T-cell responses in humans with no HLA-II- or HLA-E-restricted components [76,77] indicating a need for more complex additional vector engineering.

## 10. Safety Concerns and Attenuation

Some appealing CMV features are double-edged and linked to significant safety concerns. Therefore, HCMV persistence and latency come with the potential for regular reactivations, chronic inflammation and immune modulation. HCMV can cause severe disease in, but is not limited to, immunocompromised individuals such as those living with HIV-1/AIDS, organ transplants or cancer [78]. This may affect the deployment and effectiveness of vaccines. Persisting vaccines can be shed and transmitted through bodily fluids. HCMV can impact pregnant women and newborns due to congenital infection, which can lead to severe developmental disabilities [79,80,81]. Safety concerns have also been raised regarding a potentially tumorigenic ability of CMV following reports of detected viral DNA, mRNA and antigens in various types of tumours, opening the possibility that CMV may play a role in tumour aetiology [82,83].

These concerns may demand attenuated or replication-deficient HCMV vaccine vectors. Two laboratory strains of HCMV, Towne and Toledo (AD169), were attenuated by extensive passages through fibroblast cells and could represent a suitable starting point for further vaccine vector engineering [73,75,76]. In this respect, it was shown that while spread-deficient RhCMV68-1 vectors successfully retained the protective response [14,84], other vector attenuations could result in the loss of their ability to induce MHC-E-restricted CD8^+^ T-cell responses and control SIV [77]. It is also unclear whether or not a successful anti-HIV-1 HCMV vaccine must induce sustained IL-15 signalling. Solving these challenges on the path to an effective and safe HCMV-based vaccine platform will require improvements grounded in human data, informed by multiple small iterative experimental medicine trials.

## 11. Human Trials

The first HCMV-derived prototype anti-HIV-1 vaccine candidate tested in humans was VIR-1111. The trial took place in the US between 28 December 2020 and 5 December 2022. It was a phase 1a, first-in-human study in which 27 healthy adult participants who were considered to be at low likelihood of HIV-1 acquisition and were seropositive for HCMV received two doses of VIR-1111 expressing the Mfuse1 immunogen or placebo subcutaneously on days 1 and 57. The primary and secondary objectives were the safety and immunogenicity of the VIR-1111 vaccine; however, no study results were posted on ClinicalTrials.gov for this trial (NCT04725877) or published. As of December 2024, VIR Biotechnology, Inc, the vaccine developer and trial sponsor, is yet to release information regarding why the trial was stopped prematurely.

The second and current trial started on 19 September 2023, and the estimated completion is in November 2027. The trial is entitled “A Phase 1, Randomised, Double-Blind, Placebo-Controlled Clinical Study to Evaluate the Safety, Reactogenicity, and Immunogenicity of the HCMV-HIV Vaccine Candidate VIR-1388 in Adult Participants with Overall Good Health and Without HIV” (NCT05854381). It is multisite in the US and South Africa, active, and not recruiting. The candidate VIR-1388 vaccine was informed by experience with VIR-1111 and expresses the same HIV-1-derived Mfuse1 immunogen delivered in two subcutaneous doses on days 1 and 85. An estimated 95 volunteers will be enrolled concurrently into one of three dose levels of VIR-1388 or placebo. The overall study consists of Part A and Part B. Part A will be a lead-in phase enrolling HCMV-seropositive individuals of non-childbearing potential with a frequent safety monitoring schedule. Part B will expand enrolment into a broader population of HCMV-seropositive volunteers, including individuals of childbearing potential, who are required to use two forms of contraception. There will be an optional long-term follow-up of up to three years after the first vaccine dose. According to GlobalData, the Phase 1 trial of the candidate VIR-1388 vaccine has a 70% phase transition success rate indication benchmark for progressing into phase 2 [85].

## 12. Conclusions

Despite potential difficulties in clinical translation, development of the HCMV vaccine platform offers an exciting novel approach for treating and preventing the spread of HIV-1. It was hypothesised that HCMV as a vaccine vector could elicit prolonged, self-boosted immunity, which would help maintain a high frequency of non-exhausted HIV-1-specific effector T-cells at the site of HIV-1 entry and reactivation [24]. These T-cells could produce immediate immune interception of nascent primary infection and stop early HIV-1 replication and spread or ideally prevent detectable infection [21,24]. Instead, the RhCMV68-1 vector vaccine was shown to cause replication arrest and viral clearance post-acquisition, inducing an unconventional MHC-E-restricted CD8^+^ T-cell response [17,21]. The development of a safe and effective HCMV-based vaccine platform has been riddled with challenges. As it is always the case with the search for an HIV-1 vaccine, the risks are high, but the gain would be game changing for millions of people and economies around the world.
